# A gentle introduction to pangenomics

**DOI:** 10.1093/bib/bbae588

**Published:** 2024-11-17

**Authors:** Chelsea A Matthews, Nathan S Watson-Haigh, Rachel A Burton, Anna E Sheppard

**Affiliations:** School of Agriculture, Food and Wine, Waite Campus, University of Adelaide, Urrbrae, South Australia 5064, Australia; Australian Genome Research Facility, Victorian Comprehensive Cancer Centre, Melbourne, Victoria 3000, Australia; South Australian Genomics Centre, SAHMRI, North Terrace, Adelaide, South Australia 5000, Australia; Alkahest Inc., San Carlos, CA 94070, United States; School of Agriculture, Food and Wine, Waite Campus, University of Adelaide, Urrbrae, South Australia 5064, Australia; School of Biological Sciences, University of Adelaide, Adelaide, South Australia 5005, Australia

**Keywords:** pangenome, reference bias, genomic variation, presence–absence variation (PAV)

## Abstract

Pangenomes have emerged in response to limitations associated with traditional linear reference genomes. In contrast to a traditional reference that is (usually) assembled from a single individual, pangenomes aim to represent all of the genomic variation found in a group of organisms. The term ‘pangenome’ is currently used to describe multiple different types of genomic information, and limited language is available to differentiate between them. This is frustrating for researchers working in the field and confusing for researchers new to the field. Here, we provide an introduction to pangenomics relevant to both prokaryotic and eukaryotic organisms and propose a formalization of the language used to describe pangenomes (see the Glossary) to improve the specificity of discussion in the field.

## Introduction

**Figure 1:**
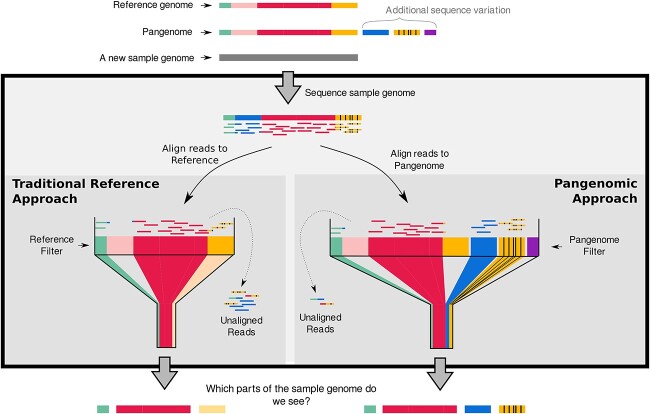
Comparison of a traditional reference approach with a pangenomic approach. In the traditional reference approach, reads from some parts of the sample genome are not similar enough to align to the reference, and so, these regions are excluded from the comparison (the blue sequence on the left). In other areas, reads will align poorly (as in the case of the orange region on the left) and so are partially or poorly represented. On the right-hand side, we can see how a pangenomic approach results in a higher proportion of reads aligning [5–7], and so, a larger portion of the sample genome is able to be analysed.

It has been established that the traditional single linear reference genome does not, and cannot, represent the full complement of genomic variation that naturally occurs within a species [1, 2]. This is a problem because many bioinformatics analyses make comparisons between a new sample and the reference sequence through read alignment. If the reference doesn’t contain a genomic sequence that is similar to the sample sequence, reads from the sample will align poorly or won’t align at all (see blue and orange regions in Fig. 1). When this occurs, the genomic sequence of the sample that’s represented by the unaligned reads is not included in the analysis. This applies to not only completely novel sequences but also regions with high allelic diversity, like the human MHC complex. Here, the sample sequence may be different enough from the reference haplotype that reads don’t align, despite this region of the genome being represented in the reference. This effect is called reference bias, and it has a significant impact on research findings [2–4]. The solution to this problem is an approach that represents more of the natural genomic variation within a species than a single linear reference genome, and this field is called pangenomics.

**Pangenomes** represent the genomic variation naturally found within a **population**, commonly a species. They may be **gene-oriented** and model the presence and absence of genes within the population, or they may be **sequence-oriented** and focus on the variation of genomic sequence including single-nucleotide variants, insertions, deletions, and structural variants for the given population. While pangenomes are often constructed at the species level, we can also build pangenomes for specific populations such as for cells within a single tissue or broader populations such as a species, phylogenetic clade, or an ecological community [8]. Pangenomes are less biased than traditional linear reference genomes [8] and have a range of applications including in species delineation, improving variant identification and genotyping accuracy, linking genes with phenotypes of interest, and inferring the haplotypes of newly sequenced samples.

Getting started in pangenomics is not straightforward. This is primarily because we don’t have a set of universally recognized descriptive terms for different types of pangenomes and their construction methods. This lack of distinction is well illustrated by Golicz *et al.*’s review of pangenome applications where they determine that they would focus ‘predominantly on studies that have been identified as pangenomic by the authors and aimed to estimate the size of the **core genome** and **accessory genome**’ [9]. The fact that a sentence was required to specify what type of pangenome they were interested in reveals a significant shortcoming in pangenomic nomenclature. This leads to difficulty in filtering the literature and makes identifying comparable analyses inconvenient for researchers working in pangenomics [10, 11]. In addition, it makes the barrier for entry into pangenomics unreasonably high.

This review has two aims. Firstly, we aim to improve the specificity of the discussion around pangenomics by formalizing the language used to describe different types of pangenomes and their construction methods. Secondly, we aim to provide a resource that will help biologists and bioinformaticians alike get a foothold on the basics of pangenomics. We do not intend to present a thorough literature review of articles applying pangenomic techniques or to give detailed instructions on constructing pangenomic models. Instead, we present the main components of pangenomics in a simplified format. We aim to instil a basic understanding of what a pangenome is, what the different types of pangenomes are, how they are generally constructed, and the types of problems they can be used to solve. We then explore some of the limitations of pangenomes and areas where research is ongoing as pangenomic models and techniques are very much still evolving.

## Definition of a pangenome

The term pangenome is used in two different contexts with subtle differences in meaning. The first is in a biological context. Within the DNA of any species or group of organisms, there is natural variation, and a pangenome used in this context refers to all of this variation. We can think of it as the complete set of genomic information for a group of organisms. However, in order to understand and make use of this variation, we need a way to capture and catalogue it. This brings us to the second use of the term—a pangenome constructed from genomic sequencing data using computational tools. In this sense, a computational pangenome catalogues the variation comprising the biological pangenome. Unless explicitly stated otherwise, the term ‘pangenome’ will be used in the context of the second meaning, the computational pangenome, for the remainder of this paper.

## Types of pangenomes

While the biological definition of a pangenome includes all genomic differences between all organisms, the full extent of this variation is inaccessible in that we can’t sequence every single organism. Even with a restricted number of sequenced organisms, the amount of variation, particularly in larger eukaryotes, can be impractical to represent and analyse efficiently. A pangenome therefore includes only a subset of this variation in order to maintain functionality.

The term ‘pangenome’ was first used in 2000 when Sigaux used it to describe a database of genome and transcriptome alterations observed in tumours, normal cells, and experimental models [12]. The pangenome Sigaux referred to was sequence-oriented in that it was a catalogue of genomic differences at the nucleotide level. In 2005, Tettelin *et al*. used the same term to describe the presence and absence of genes within eight *Streptococcus agalactiae* genomes [13]. Tettelin *et al*.’s pangenome was gene-oriented in that it detailed the presence or absence of entire genes within the population with a focus on gene function.

**Figure 2:**
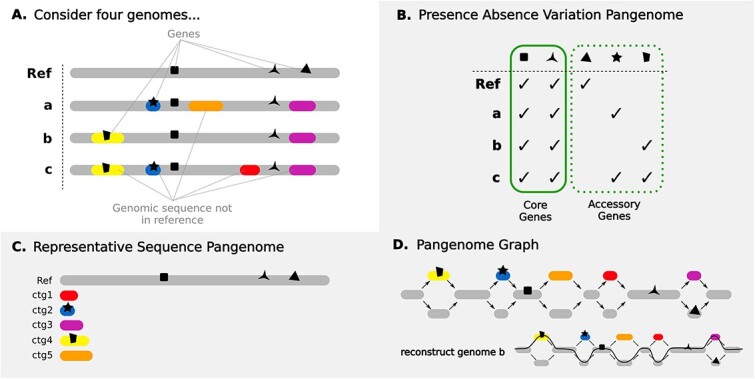
Three types of pangenomic data structures. (A) Consider a collection of four genomic sequences—one reference genome and three other genomes from the same population. Coloured sections indicate regions in genomes a, b, and c that diverge from the reference. Genes are indicated by black shapes. (B) PAV pangenome. Genes found within a population are partitioned into two groups: the core genome, which includes genes present in all members of the population, and the accessory genome, which includes genes present in only some members of the population. (C) A representative sequence pangenome. A set of genomic sequences such that the bulk of sequence diversity from the population is represented without significant duplication. (D) A sequence-oriented pangenome graph. A graph structure composed of nodes (genomic sequence) and edges (arrows between the sequence). Specific paths through the graph correspond to haplotypes present in the population. Pangenome graphs may also be gene-oriented, in which case each node represents a gene and edges indicate gene adjacency in the input genomes (see Fig. 3 for more details).

**Figure 3:**
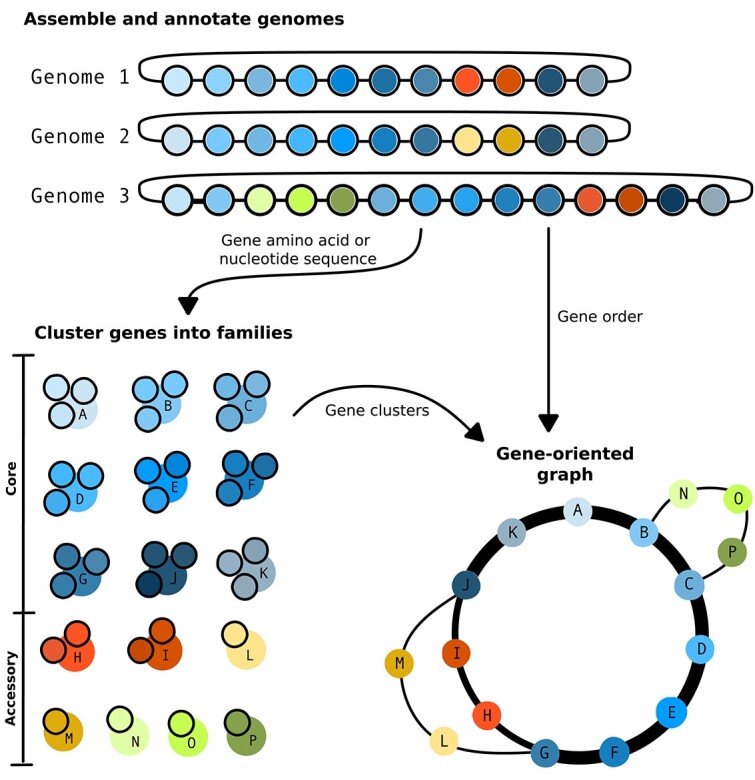
Gene-oriented graphs. Genomes are assembled and annotated, and the amino acid sequence or the nucleotide sequence of all genes is extracted and clustered. Each cluster makes up a single node of the graph, and the lines between the nodes connect genes that are adjacent in the input genomes. The thicker the line, the larger the number of genomes that have these two genes adjacent. If we consider Genome 1 to be the reference genome in this example, then Genome 2 has genes L and M (in yellow) instead of the reference H and I (in red) while Genome 3 has an insertion of the genes N, O, and P (in green) between genes B and C.

To aid researchers in the identification of relevant literature, we identify three major types of pangenomes (Fig. 2). The first is a ‘presence–absence variation pangenome’ (PAV, originally described by Tettelin *et al.* [13]), which is composed of a ‘core genome’ and an ‘accessory genome’. The core genome is the full set of genes that are present in every member of the population, while the accessory genome is composed of genes present in a subset of the population. As the name suggests, this type of pangenome focuses on gene presence and absence; it does not account for gene location, allelic diversity of genes, or intergenic sequence (Fig. 2B). The second type of pangenome is a ‘representative sequence pangenome’. This type of pangenome is composed of carefully selected genomic sequences so that as much genomic variation from the population as possible is represented using as little sequence as possible. It has the same structure as a traditional reference genome but with additional contigs containing supplementary genomic sequences (Fig. 2C). The third type of pangenome is a ‘pangenome graph’, sometimes called a graphical pangenome. A pangenome graph may be sequence-oriented or gene-oriented. A sequence-oriented pangenome graph models genomic sequence variation as well as its location relative to other genomic sequences from the population (see Fig. 2D), while a gene-oriented pangenome graph models the genes found within the population and their order relative to other members of the population (see Fig. 3).

## Presence–absence variation pangenomes

PAV pangenomes (Fig. 2B) quantify the presence and absence of genes within a population. They identify the core genome, which includes all of the genes present in all members of the population, and the accessory/dispensable genome, which includes all of the genes present in a subset of the population [13]. Core gene functions are generally under high selective pressure and are highly conserved within the population. They tend to be older [14] and essential for survival, while accessory genes tend to be less conserved and responsible for variations in lifestyle and evolutionary trajectories [13]. This is not to say that the core genome is the minimal set of genes required for the organism to survive and thrive. Rather, it can be thought of as ‘a backbone of essential components on which the rest of the genome is built’ [15].

### Construction

There are two main strategies for constructing a PAV pangenome: the **homolog-based strategy** and the **map-to-pan strategy** [16].

#### Homologue-based strategy

In the homologue-based strategy [16], *de novo* assembled genomes are annotated individually, and the nucleotide or amino acid sequence of each protein coding gene is extracted. The sequences are pooled and then clustered into groups based on their sequence similarity, usually based on Basic Local Alignment Search Tool (BLAST) alignments, but alignment-free methods may also be used [17, 18]. This clustering step is often referred to as ‘homology clustering’ [16] or ‘orthologous gene detection’ [17]. Many pangenome analysis tools also have the option to split clusters further, enabling the separation of paralogous genes, though these strategies vary between tools and give differing results [18]. Clusters that contain a sequence from every member of the pangenome are considered core genes, while clusters comprised of gene sequences present only in some members of the pangenome are considered accessory genes.

The homologue-based strategy is based on sequence clustering. Because of this, the overall pangenome size and the core genome are very sensitive to the sequence identity and sequence coverage parameters chosen [19]. If these values are set too high, orthologous genes may be mistakenly split into multiple clusters, and the number of genes within the pangenome will be overestimated [19]. The inverse is also true. If the sequence similarity and sequence coverage thresholds are set too low, nonorthologous genes can be clustered together causing the total size of the pangenome to be underestimated and possibly overestimating the size of the core genome.

The homologue-based strategy is most widely used in bacteria [16] because their simple gene structure and small genome size make them much easier and cheaper to annotate in large numbers than eukaryotic genomes, which tend to be much larger and where genes often contain introns. Despite these challenges, this method has still been applied in some eukaryotes [20, 21]. To reduce the complexity of this strategy, a modified method has emerged whereby only a subset of assemblies comprising the pangenome are annotated. Gene models from these assemblies are then aligned to the remaining assemblies, and orthology clustering is used to determine presence and absence of gene groups [22].

Many tools for constructing PAV pangenomes for bacteria construct a gene-oriented graph as part of their analysis workflow [23–25], though only some tools provide a way to visualize this graph [18]. These graphs model gene presence and absence as well as gene order within a group of organisms, unlike sequence-oriented graphs, which model differences in genomic sequence at the nucleotide level (see the Pangenome Graph section). They are made up of nodes and edges with each node corresponding to a single gene cluster and edges joining together nodes of genes that were adjacent in their original assembly (Fig. 3). Visualizing these graphs allows us to interact with our data in a more natural way. For example, we can identify blocks of gene synteny (samples that follow the same path through part of the graph), find potential contaminants (genes found in only a single genome), and identify patterns that would otherwise be missed [26].

#### Map-to-pan strategy

In the map-to-pan strategy, a gene is determined to be present in a sample by aligning whole-genome sequencing reads to an annotated representative sequence pangenome. Because the likelihood that any region of the genome is covered by reads is dependent on the total sequencing read depth, a minimum total read depth for the genome of 10× is recommended to reduce the likelihood that a gene will be incorrectly found as absent [27, 28]. A threshold of 10× allows for ~99% recovery of gene presence [29, 30]. With a minimum sequencing depth of 10×, a minimum of 5% coverage of a gene’s exons from at least one read is widely used to determine that a gene is present [27, 29, 31], but this threshold may be higher where the minimum sequencing depth for the genome is higher. For example, Wang *et al.* [14] used a minimum sequencing depth of 20× to determine gene presence and absence and determined that a gene was present when 95% of its exons were covered by reads and 85% of the gene body was covered. As in the homologue-based strategy, genes that are found to be present in all samples comprise the core genome, while genes found in only one or some samples make up the accessory genome.

#### Expanding our definition of ‘core’

Core genome classification is very sensitive to technical artefacts. If a true core gene is erroneously not identified in just one sample, it isn’t classified as being part of the core genome. To account for some of this uncertainty, some analyses allow that core genes be present in <100% of samples (Lapierre and Gogarten use 99% [15]), or they define an additional ‘softcore’ genome that contains genes present in a high percentage of samples, often 95% or greater [32–34]. The remaining accessory genes may also be split into a shell genome (genes present in many genomes, e.g. 1%–99%) and a cloud genome (genes present in very few genomes, e.g. <1%) [30, 35].

#### The intersection of pangenomics and metagenomics

Metagenome-assembled genomes (MAGs) are genomes assembled from genomic sequencing of samples composed of multiple different microorganisms. The metagenomics approach bypasses the need for individual species isolation and cultivation, allowing us to study the composition and interactions of microbial communities. However, it results in genome assemblies that are often fragmented or incomplete. The application of pangenomic methods to MAGs allows us to explore the genetic composition of these communities, but using traditional methods results in significant loss of core genes due to MAG fragmentation [36]. There are a range of techniques that can be used to alleviate this, the simplest of which is lowering the core gene occurrence frequency threshold [36]. Other approaches ‘infer’ the presence or absence of genes, for example, by selecting from a set of complete genomes the most similar complete genome to the incomplete/fragmented genome and using it as a reference to infer the remainder of the incomplete genome [37].

### Applications

PAV pangenomes allow us to investigate the pattern of gene presence and absence within a species or other phylogenetic clade. This improves our understanding of genotype–phenotype associations and gives evolutionary insights [38, 39]. The very first PAV pangenome was constructed from eight strains of *S. agalactiae* bacteria. It enabled the development of a protective vaccine [40] and led to the functional characterization of important genetic determinants [41]. This type of pangenomic analysis has been used widely in prokaryotes, contributing to the identification of genetic signatures for antibiotic resistance [42], genes associated with pathogenicity [43, 44], and possible drug targets [45, 46]. Pangenomes have also been used in agriculture to support crop improvement through the identification of genes lost from the germplasm through domestication and breeding that are still present in wild relatives [30, 47–49]. By comparing the core genome of a wild species and the accessory genome of a cultivated species, we can see the impact of domestication and can then work towards breeding any lost desirable traits back into the germplasm [20, 30, 50]. PAV pangenomes also have applications in phylogenetics. For example, Gaba *et al.* used the Halobacteria core genome to inform a multigene approach to phylogeny inference where a traditional single gene approach was inappropriate due to significant sequence divergence [34].

## Representative sequence pangenome

Representative sequence pangenomes are a collection of genomic sequences that minimize the inclusion of homologous loci while still representing as much genomic diversity from the population as possible. They are usually composed of a reference genome and a number of other sequences called nonredundant reference (NRR) sequences. NRR sequences are sequences that are found in at least one member of the population but are not represented in the reference.

### Construction

**Figure 4:**
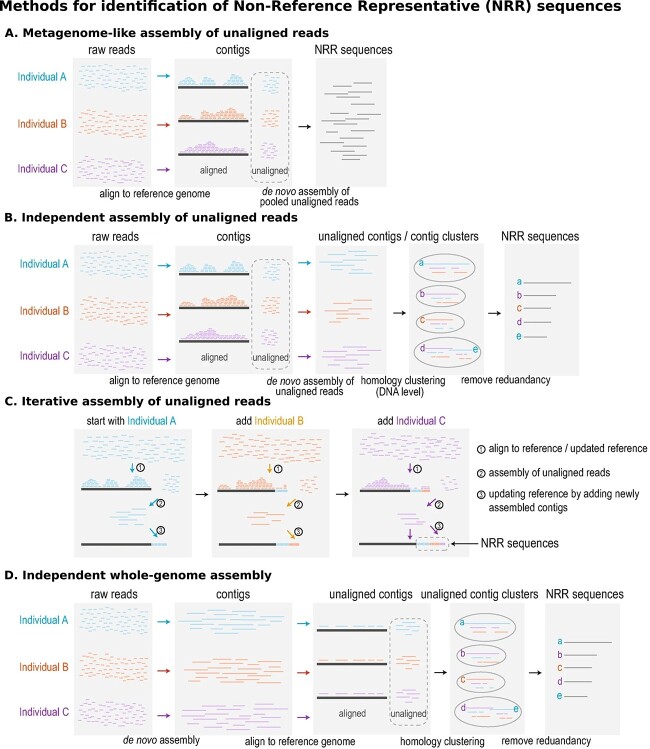
Four methods for identifying NRR sequences. Adapted from [16] (https://creativecommons.org/licenses/by/4.0/). (A) Reads from all samples that don’t align to the selected reference genome are pooled and *de novo* assembled into NRR sequences. (B) For each sample, reads that don’t align to the reference genome are *de novo* assembled into contigs. All contigs are pooled and then clustered to remove redundant sequences. (C) Reads that don’t align to the reference genome are *de novo* assembled into contigs, and the reference genome is updated to include these contigs. This process is repeated iteratively for all samples with the reference genome growing incrementally. (D) All reads for each sample are *de novo* assembled into contigs. Contigs are aligned to the reference genome and all unaligned contigs are pooled for all samples. Clustering is then used to remove redundant sequences.

A representative sequence pangenome is constructed by identifying genomic sequences that aren’t already present in the reference genome. These sequences are appended to the reference genome as additional contigs to form a pangenome reference. The pangenome reference may then be optionally annotated. There are four different methods for the identification of NRR sequences [16] termed metagenome-like assembly of unaligned reads, independent assembly of unaligned reads, iterative assembly of unaligned reads, and independent whole-genome assembly. These methods are briefly described below (see Fig. 4).

#### Metagenome-like assembly of unaligned reads

Reads from all samples are aligned to the reference genome, and unaligned reads are collected, pooled, and *de novo* assembled [51] (Fig. 4A). The contigs resulting from *de novo* assembly are the NRR sequences, which are then appended to the reference genome. This method works well even with very low read coverage (as little as 1× coverage if the total number of samples is very high [30, 51]).

#### Independent assembly of unaligned reads

Reads are aligned to the reference genome, and unaligned reads from each sample are individually *de novo* assembled (Fig. 4B). The resulting contigs are pooled and clustered by sequence similarity. A representative sequence is taken from each cluster and appended to the reference genome [52, 53]. This method requires at least 10× read coverage to generate contigs of sufficient size.

#### Iterative assembly of unaligned reads

In this method, the pangenome is constructed incrementally (Fig. 4C). Reads from a single sample that don’t align with the reference are *de novo* assembled into contigs. These contigs (NRR sequences) are then appended to the reference genome, and this updated reference is used for processing the next sample. This process is repeated for all samples with new contigs from each sample updating the reference.

#### Independent whole-genome assembly

Reads from each sample are *de novo* assembled separately into contigs, which are then aligned to the reference genome (Fig. 4D). Unaligned contigs are pooled and clustered by sequence similarity. The longest sequence (an NRR sequence) is taken from each cluster and appended to the reference genome to form the pangenome.

In addition to each of the methods described above, NRR sequences undergo some additional filtering before the pangenome is finalized. NRR sequences <~500 bp in length are usually excluded [14, 30, 54–57]. NRR sequences that are very similar to the reference sequence are also removed (90% sequence identity with the reference is commonly used [30, 57, 58]), and any remaining NRR sequences may be compared against the National Center for Biotechnology Information (NCBI) nt database using BLAST to remove potential contaminants [10, 57].

#### Selecting a method

Selecting a method for the construction of a representative sequence pangenome depends on a number of factors including the type of genomic data available, the quantity of data available per sample, the number of samples, and the available computing resources. In the case that only very low coverage data are available (<10×) but for a large number of samples, the metagenome-like assembly method is most appropriate. One drawback to this method is that it can result in chimeric contigs [16], which are artificial contigs not found in any sample but rather chimaeras of sequence data from at least two samples. This can be partially mitigated by partitioning samples into different species or known genetically distinct groups before *de novo* assembly [51]. In cases where higher coverage data are available (>10× coverage), either the assembly of unaligned reads or the iterative assembly approach would be suitable. Of these, the former allows the pangenome to be constructed in a parallel fashion, whereas the latter approach requires that each sample is processed one after the other. This iterative approach may take longer when the number of samples is very high but has the advantage that additional samples may be added to the pangenome with less effort at a later date. In addition, with the assembly of unaligned reads approach, the clustering step may require substantial computational resources if the sample number is very high; the iterative approach does not suffer from the same scaling limitations. In the case that multiple high-quality genome assemblies already exist, the independent whole-genome assembly method may be the best choice as the resulting NRR sequences will likely be longer. Note that these methods may be combined and modified [31, 51]. This is often done to make use of existing sequence data where some samples have very low coverage and others have much higher coverage.

##### Applications

Representative sequence pangenomes can be used in most bioinformatics workflows or analyses that would usually use a traditional linear reference genome including in variant calling, genotyping, and transcriptomics expression analyses. In these applications, a representative sequence pangenome usually outperforms a traditional reference genome [58, 59]. Representative sequence pangenomes may also be annotated in much the same way as a traditional reference genome. In this form, they can be used as the basis for a PAV pangenome constructed using the map-to-pan approach [16].

The human reference GRCh38.p14 is an example of a representative sequence pangenome in common use, though a relatively incomplete one. It is a pangenome in the sense that it is composed of a primary reference sequence and a number of ‘alternative’ contigs that contain common genomic variants not represented by the reference sequence [60]. These alternative contigs have a total length of 109Mbp and span ~60Mbp of the primary assembly [60]. However, it is incomplete in the sense that there is a huge amount of natural sequence variation not represented [61]. The recently released T2T-CHM13 assembly (a gapless telomere-to-telomere genome assembly generated from a single human) identified 182Mbp of sequence not represented by GRCh38 [62], while the Chinese human pangenome [58] and the African human pangenome [63] identified 276Mbp and 296Mbp of novel genomic sequence, respectively. In addition to this lack of representation, many read alignment tools simply don’t make use of the alternative contigs present in GRCh38 [64].

## Pangenome graph

A sequence-oriented pangenome graph models the location of genomic variation within a species with respect to either a reference sequence or to the other sequences comprising the pangenome. They are composed of a set of ‘nodes’ and ‘edges’. Nodes are segments of genomic sequence, and edges join these segments together. The basic concept of a pangenome graph is illustrated in Fig. 2D.

### Construction

**Figure 5:**
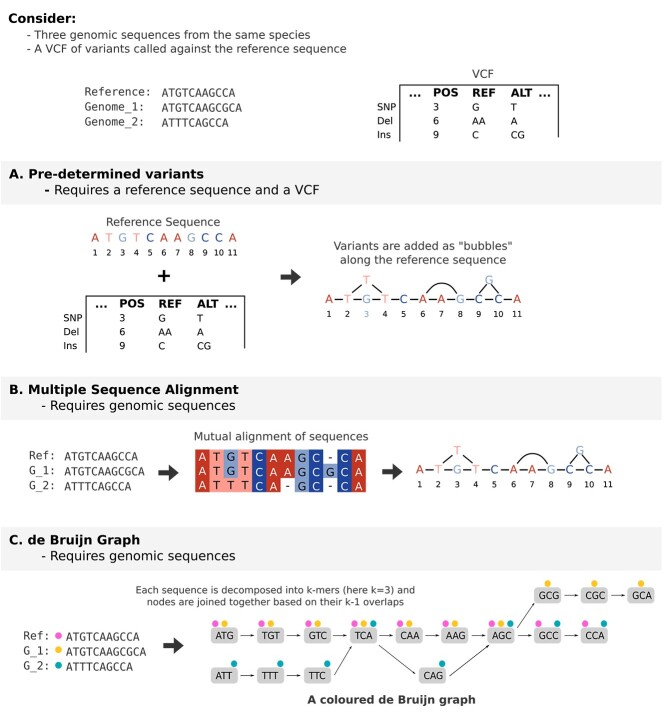
Three methods for constructing sequence-oriented pangenome graphs. (A) Variants are added to the graph as bubbles ordered along the reference sequence. (B) Multiple genomic sequences are aligned to each other by introducing spaces into their sequences so as to maximize the number of bases that match up at each location. (C) A de Bruijn graph is constructed by breaking all genomic sequences up into *k*-mers, creating nodes from all *k*-mers that appear at least once, and connecting nodes that overlap each other by *k*-1.

There are three main methods for constructing sequence-oriented pangenome graphs (see Fig. 5).

#### Predetermined variants

This method (Fig. 5A) requires a reference genome/sequence and a set of predetermined variants. Using a reference as the base of the graph, each variant is added to the graph as a ‘bubble’, resulting in a directed acyclic graph ordered along the reference genome. This method is used by a number of popular tools [6, 11, 65, 66] and is one of the more common methods of graph construction, particularly for species for which there is a single high-quality linear reference genome already available [67].

#### Multiple sequence alignment

Pangenome graphs may also be constructed through the alignment of genomic sequences directly against each other, termed multiple sequence alignment (Fig. 5B) [67–70]. While this is computationally very expensive when compared with the predetermined variants method above, it is particularly useful in cases where multiple high-quality sequences or phased assemblies are already available as this method enables the graph to retain haplotype/phasing information [71].

#### De Bruijn graphs

De Bruijn graphs (Fig. 5C) were historically applied to short-read genome assembly but have since been used in pangenome graph construction, among other applications. Briefly, reads are split into *k*-mers (subsequences of the read with length *k*) to form the nodes of the graph, and nodes are joined together based on their *k*-1 sequence length overlap with one another. Intuitively, the decomposition of input reads into *k*-mers means that long-range connectivity is lost, but techniques have been developed that reconstruct these input sequences [72, 73]. The de Bruijn graph pangenome can be extended by colouring *k*-mers that originate from the same sample [74] and by ‘compacting’ the de Bruijn graph whereby adjacent nodes connected by a single path are condensed into a single node with a longer sequence label [75]. These properties mean that multiple genomes can be assembled simultaneously [75–77], and their construction essentially combines variant detection, *de novo* assembly, and graph construction into a single algorithm [77] that additionally doesn’t require a reference.

### Applications

The applications of pangenome graphs centre around their ability to improve mapping accuracy and hence reduce reference bias [78]. In particular, they have applications in genotyping, haplotype inference, and functional genomics.

#### Genotyping and variant calling

Pangenome graphs improve the accuracy of variant genotyping with short reads marginally for small variants [79] and more significantly for large structural variants (variants >50 bp in length). This is because short reads span the entirety of small variants but are not able to span larger structural variants. Long reads are better suited to this task; however, they are more expensive to generate and much less prevalent than short reads in current public datasets. By using a pangenome graph containing known structural variants, short-read coverage along the graph can be used to genotype structural variations with more accuracy than traditional short-read methods using a single linear reference genome [70, 78]. Furthermore, Ebler *et al.* found that a graph constructed from existing haplotype-resolved assemblies (a multiple sequence alignment pangenome graph) could be used to genotype structural variants using *k*-mer distributions from short reads that were inaccessible to other short-read variant calling methods [80]. Where a reference genome is not available, de Bruijn pangenome graphs enable reference-free structural variant calling [77].

#### Haplotype inference

Most diploid or polyploid genomes are represented by a haploid assembly whereby homologous chromosomes are compressed into a single haploid representation. This results in the loss of heterozygous variation as well as errors where haplotypes vary. Haplotype inference—or the phasing of assemblies—ensures that the variation between different copies of the genome is preserved. By constructing a pangenome graph from the multiple sequence alignment of high-quality phased reference sequences and augmenting the resulting graph with existing catalogues of variation, Dilthey *et al.* were able to infer diploid personalized reference genomes for new samples based on short reads alone [81].

#### Functional pangenomics

Using a pangenome in the place of a traditional linear reference can reduce errors in comparative gene expression estimation. For example, in allele-specific expression analysis, a mapping bias between alleles of interest can alter comparative gene expression estimates [82]. By using a pangenome that incorporates additional variation and better represents the alleles of interest, mapping bias can be reduced or eliminated and comparative gene expression can be more accurately estimated.

### Graph pangenome challenges

Linear reference genomes use a coordinate system to track the locations of many genomic features including genes and variants. Using a simple linear numbering system, these coordinates tell us exactly where along the length of the reference genome a feature is located. This coordinate system is easy to interpret and unambiguous, but the convenience of this approach doesn’t translate to the more information-rich pangenome graph [83]. There are multiple different paths through a graph and, hence, different lengths of genomic sequences. This means that bases can no longer be numbered sequentially because their distance from the start of the pangenome depends on the starting point and which path was taken through the graph. There have been a number of proposed solutions, but none have been universally adopted [64, 74, 79, 83].

The utility of pangenome graphs lies in the way that they model the adjacency of different sequences, but this structure makes them very difficult to visualize, particularly with respect to annotations. Where a linear sequence (a traditional reference genome or a representative sequence pangenome) can be easily depicted on a screen with annotations labelled under the sequence, a pangenome graph cannot be visualized this way. Tools for graph visualization tend to either focus on the larger graph structure [84, 85] or on the base-level structure [70, 86], and moving between these scales, particularly for larger eukaryotic genomes, remains an open problem [79].

## Considerations

### Open versus closed pangenomes

**Figure 6:**
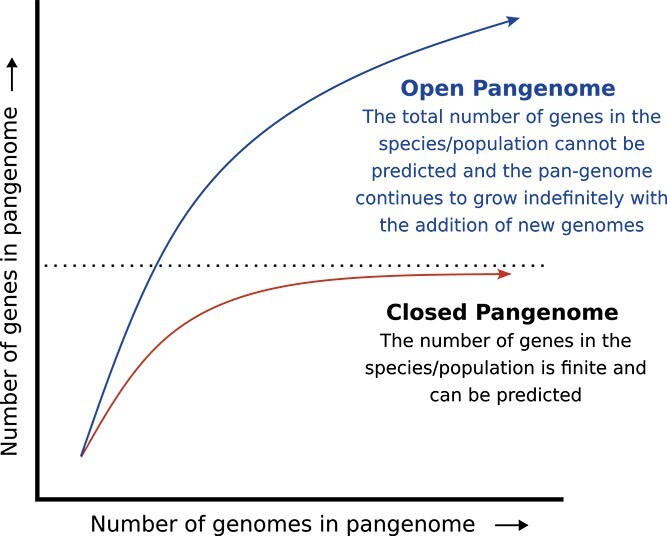
Open and closed pangenomes. As the number of genomes included in the pangenome increases, the total number of genes in the pangenome will either plateau (a closed pangenome) or will continue to increase so that the total number of genes for that species/population cannot be accurately estimated (an open pangenome).

PAV pangenomes can be classified as being either ‘open’ or ‘closed’. An open pangenome implies that the number of genes added to the pangenome doesn’t diminish with the addition of each new genome and that it is not possible to determine how many genomes would be needed to identify all genes within the species [87] (see Fig. 6). This is often the case for sympatric bacteria where genetic material is readily exchanged between species, thereby continually growing the gene pool [88]. *Escherichia coli* [89, 90], *S. agalactiae* [13], and *Pseudomonas aeruginosa* [90] have all been found to have open pangenomes.

On the other hand, for a species with a closed pangenome, the addition of new genomes provides diminishing returns (see Fig. 6). The pangenome grows more slowly with the addition of each sample and eventually stops growing when the entire gene repertoire for that species is represented. Soybean [20, 55], *Brassica oleracea* [10], and *Staphylococcus lugdunensis* [91] have all been found to have closed pangenomes.

### Sampling strategy

An effective sampling strategy for pangenome construction helps to manage costs and maximize the inclusion of genomic diversity. In agriculture, a collection of as few samples as possible that maximizes the inclusion of genetic diversity is known as a core collection [92], and this collection can be surprisingly small with as little as 5% of a population retaining ~90% of genetic variation [93, 94]. As a rule, species with a closed pangenome will require fewer samples than species with an open pangenome. Strategies and software for selecting samples for inclusion in a core collection include consideration of geographical location, taxonomic classification, morphological characteristics, molecular markers, and combinations of these strategies [94–96]. Neglecting to employ a suitable sampling strategy will result in resource wastage and, when sufficient samples are not included, the identification of fewer genes/unique sequences as well as the artificial inflation of the core genome.

### Variation selection

Including too much variation in a representative sequence pangenome or a pangenome graph can actually reduce the utility of the pangenome [97, 98]. This is particularly true for eukaryotes with large genomes. When the sequences comprising the representative sequence pangenome are very similar to one another, reads will map to multiple locations or (depending on the aligner) won’t map at all [97]. Similarly, in a pangenome graph, the incorporation of too much variation increases instances of read multimapping and reduces alignment accuracy [98] but with the additional concern of ‘blowup’ where the computational cost of alignment becomes so high that the graph becomes unusable [97]. Therefore, criteria for variant inclusion in a pangenome should be considered. In a representative sequence pangenome, this can be as simple as carefully examining clustering parameters and sequence similarity/sequence identity thresholds to ensure that NRR sequences are sufficiently different from each other and the reference. In a pangenome graph, this could involve prioritization of more common variants/alleles for that particular population [99] or using a tool specifically designed to select variation that maximizes the utility of the graph [97]. The decision of which variation to include in a pangenome graph is nontrivial and is an active area of study [97].

### Where to go from here

In prokaryotic and small eukaryotic pangenomics, PAV pangenomes are most commonly used, and many tools are available for this type of analysis (see [18] for a comparison of 16 different tools). The selection of a method and parameters for gene clustering has a significant impact on the resulting pangenome (see [100] for discussion on this topic), and, despite their wide application, many challenges remain in prokaryotic pangenomic analyses (summarized in [101]). Tools that automate the construction of representative sequence pangenomes and PAV pangenomes in larger eukaryotes [102–104] are less numerous, and most studies still use *ad hoc* pipelines [20, 21, 54, 105, 106]. Methods and tools for constructing sequence-oriented graph pangenomes are also still under active development, but some details and comparisons of methods can be found in [107–110].

## Closing statement

Pangenomes represent the genomic content of a population much more completely than a traditional single linear reference genome. They are less restrictive than a traditional reference in that they can be used in the analysis of phylogenetic clades above the species level, and they improve research outcomes by reducing reference bias. While the tools that we use to construct and interact with pangenomes, particularly graph pangenomes, are still under development, they have proven their worth and have begun to make their way into mainstream analysis pipelines [71]. This transition will be aided by individual and laboratory efforts as well as large international consortia including the Human Pangenome Reference Consortium and the Computational Pan-Genomics Consortium [8], and we believe that the formalization of language used to describe pangenomes will contribute to the effective dissemination of research surrounding them.

**Key Points**

- Pangenomes can be constructed in different ways, from different types of genomic data, and for different purposes.
- Pangenomes aim to represent the full genomic repertoire of a population, not just the genome of a single individual.
- In comparison with traditional reference-based techniques, pangenomes greatly reduce reference bias.
- The field of pangenomics is quickly growing but is complex, and the language used in the description of pangenomic methods lacks specificity.
- We introduce pangenomics for a newcomer to the field and provide suggestions to formalize the language used in the discussion of pangenomes.

**Glossary of useful terms**

**Accessory genome**—a set of genes that are present in only one or some members of a population.

**Core genome**—a set of genes that are present within all members of a population.

**Gene-oriented pangenome**—a pangenome modelling differences within a population at the gene level.

**Homologue strategy**—multiple genomes from the population are annotated, and gene sequences are extracted from these annotations and are clustered by sequence similarity into gene clusters; the presence or absence of a gene in a sample is determined by whether or not that sample contributes to that gene cluster.

**Map-to-pan strategy**—determination of gene presence through interrogation of sample read coverage of genes.

**Pangenome**—genomic data from multiple members of a species or other population with some underlying structure.

**Pangenome graph**—a pangenome that is represented using a mathematical graph structure composed of nodes and edges that gives positional context to sequence variation between different genomic sequences.

**Population**—a group of organisms that may benefit from genomic comparison, for example, a specific tissue, species, phylogenetic clade, or ecological community.

**Presence**–absence variation pangenome—a collection of all of the genes found within a population that is divided into a core genome and an accessory genome based on gene patterns of presence and absence within the population.

**Representative sequence pangenome**—a collection of genomic sequences that, together, represent the majority of natural sequence variation within a population.

**Sequence-oriented pangenome**—a pangenome modelling differences within a population at the sequence/nucleotide level.

## Data Availability

No new data were generated or analysed in support of this research.
